# Studies of Potential Migration of Hazardous Chemicals from Sustainable Food Contact Materials

**DOI:** 10.3390/foods13050645

**Published:** 2024-02-21

**Authors:** Giulia Simonetti, Carmela Riccardi, Donatella Pomata, Luca Acquaviva, Andrea Fricano, Francesca Buiarelli, Marta Senofonte, Patrizia Di Filippo

**Affiliations:** 1 Department of Chemistry, Sapienza University of Rome, 00185 Rome, Italy; giulia.simonetti@uniroma1.it (G.S.); luca.acquaviva1@libero.it (L.A.); andrea.fricano@uniroma1.it (A.F.); marta.senofonte@uniroma1.it (M.S.); 2 Italian Workers’ Compensation Authority (INAIL)—DIT, 00143 Rome, Italy; ca.riccardi@inail.it (C.R.); d.pomata@inail.it (D.P.); p.difilippo@inail.it (P.D.F.)

**Keywords:** bio-based food contact materials, migration of toxic compounds, PFASs, OPEs, HPLC-Q-Trap

## Abstract

In recent years, due to modern techniques for the distribution, transport, and retail sale of food, the production of large amounts of non-biodegradable and bioaccumulative packaging waste has become a major environmental issue. To address this issue, new food packaging materials based on renewable biomass have been studied as eco-friendly, biodegradable, and biocompatible alternatives to synthetic materials. However, although these materials are not petrochemical derivatives, the presence of contaminants cannot be excluded. This work aims to extend the knowledge on bio-based packaging materials, researching the presence of contaminants potentially able to migrate to food at concentrations of concern. In this study, we focus on two classes of contaminants, organophosphate esters (OPEs) and perfluoroalkyl substances (PFASs), carrying out migration tests toward different simulants, according to the current European regulation. PFAS analysis was performed using high-resolution liquid chromatography coupled to ion trap-tandem mass spectrometry (QTrap). OPE analyses were performed both by gas chromatography–mass spectrometry (GC-MS) and high-resolution liquid chromatography coupled to triple quadrupole mass spectrometry (TQMS). Preliminary findings demonstrate the release of toxic OPEs and PFASs from bio-based food packaging, highlighting the need to investigate the presence of potentially harmful chemicals in these materials.

## 1. Introduction

In order to reduce environmental impact and dependence on fossil fuels, the packaging and containers industry has recently started to move toward the production of sustainable and green materials derived from renewable biomass: the so-called “Bio-Based” Food Contact Materials (BBFCMs) [1,2,3]. Among the environmentally friendly alternative materials, plant fiber products have received particular attention [4]. This sort of materials often ensures effective disposal and/or recycling processes that can help protect the environment; however, it is mandatory for them to be equally safe for human health.

Although there is no specific regulation for these materials, Regulation 1935/2004/EC [5] applies to any food contact material (FCM), stating that the use of any substance that is harmful to human health and potentially transferable to food is not authorized for FCMs production of or as an additive.

Therefore, the study of any substance that can be transferred to food from the container, potentially present in FCMs, is of great importance [6]. 

From a hygiene point of view, compounds that can migrate from the “bio-container”, or any other “biomaterial” in contact with the food during the various processing stages, must be avoided. Such substances can in fact increase the risk of chemical contamination of the food itself, posing a risk to human health. 

Rather than the constituents of a container themselves, it is interesting to know whether any of these constituents are likely to be transferred to the food through contact and to what extent.

Therefore, a toxic substance, which cannot be added to food as an additive, may be used as a technologically irreplaceable constituent of a food container under the condition that it does not migrate to the food itself. 

The possibility of food contamination by contact transfer depends on three parallel factors:

(a) Temperature and contact duration;
(b) Nature and chemical–physical characteristics of the material in contact with the food;
(c) Nature of the food that interacts with the container [7].

To ascertain the suitability of a container under the real usage conditions, it would be best to apply migration tests to packaged food. However, since the detection and quantitation of traces of contaminants in such a complex matrix would be analytically challenging, liquid food simulants represent a more appropriate solution.

According to the current legislation [8], specifically on “plastic materials and articles intended to come into contact with food”, migration tests consist of keeping the container in contact, under controlled time and temperature conditions, with a pure solvent chosen accordingly to the nature of the food to be packaged or that is supposed to interact with the investigated material [9]. 

Eventually, the global migration of contaminants or the specific migration of single compounds can be evaluated. The first is carried out by evaporating the solvent, weighing the residue, and expressing the results in milligrams/L. The overall migration allows to also identify substances that are of no concern from a hygiene point of view but whose release should be equally limited in order to avoid the adulteration of food. After the contact between FCMs and the simulant, this specific migration study can determine single substances for which particular and severe limitations stand [10].

The regulation (EU) 10/2011 [8] specifically applies to plastic multi-layer materials separated from food by a functional barrier. This regulation establishes a statutory limit value of 0.01 mg/kg in food for the migration of non-authorized substances through the functional barrier.

*This limit shall apply to groups of compounds, structurally and toxicologically related, isomers or compounds with the same relevant functional group* [8]. Mutagenic, carcinogenic, or reprotoxic (CMR) substances should not be used in FCMs without previous authorization.

The Commission Recommendation (EU) 2019/794 [11] has recently set out a control plan to establish the prevalence of substances migrating from FCMs to food; in Sample Descriptions and Methodology, a list of specific substances to be monitored features several classes of compounds such as fluorinated compounds in paper and board-based materials and articles. 

Specifically, among fluorinated compounds, PFOA has been the only PFAS restricted for use in articles, including food contact materials since 2017 [12]. However, early in 2023, a proposal to restrict or ban all PFASs from food contact materials has been submitted to the European Chemicals Agency (ECHA), since PFASs can be easily replaced with substitutes [13]. 

After an appropriate evaluation process intending to assess whether this proposal meets a favorable cost–benefit ratio, the prohibition on PFASs in the EU will likely be operative sometime in 2027 [13].

In our previous papers, we examined various baking paper and aluminum foil samples in order to evaluate the occurrence and migration to simulants of chemicals such as PFASs and OPEs, which are both emerging contaminants [14]; subsequently, we focused on bio-based FCMs to assess the migration of some new brominated flame retardants and bromophenols [15]. In the last case, we examined eco-sustainable, biodegradable, and compostable packaging materials typically used as alternatives to those derived from mineral oil-based polymers. 

The present work extends our previous studies and aims to determine the presence of emerging contaminants in sustainable BBFCMs and their migration to food simulants, allowing us to verify the wholesomeness of these materials and their safety of use. 

These molecules could be processing contaminants inserted to give functional properties to the packaging material. For example, OPEs are flame retardants and plasticizers, whereas PFASs are coating and water and grease repellents. Goossen has addressed this topic extensively in a recent study [16]. On the other hand, it cannot be excluded that due to their ubiquitous nature, they are unintentional contaminants, coming from the environment (water, soil, agriculture, air, etc.).

To this aim, release tests approved and validated by European legislation were carried out to evaluate the migration of twelve OPEs and twenty-two PFASs from different types of BBFCMs, which are all biodegradable or compostable products.

After gathering information from the scientific literature [10,17], a proper analytical procedure was developed and optimized for studying the transfer of these analytes. OPE chromatographic and mass spectrometric conditions are discussed in a previous paper [14,18]; the PFAS analysis method was developed and validated by liquid chromatography coupled to ion trap-tandem mass spectrometry. 

The number of migrated contaminants was determined using two different food simulants: 95% ethanol (D2 simulant) and 3% acetic acid (simulant B) solutions were used to simulate the behavior of fatty substances and aqueous–acidic foods, respectively. According to the directives of European regulation N°10/2011 [8], migration tests were carried out under three different sets of conditions, exposing the studied packaging materials to the simulant for 10 days at 40 °C (OM test 2), for 2 h at 70 °C (OM test 3), and for one hour at 100 °C (OM test 4). 

Results obtained for concentrations of migrated substances were compared to the limit value reported in Regulation (EU) 10/2011 (0.01 mg/kg), although it specifically refers to plastic materials [8].

The results achieved show a non-negligible migration of some emerging contaminants and encourage further and more extensive investigation to verify the effective safety of these bio-based materials.

The possible food contamination is of great concern due to the confirmed toxicity of some of these perfluorochemicals, putting human health at risk. In particular, the last EFSA scientific opinion stressed their hepatotoxicity and reproductive toxicity [19].

## 2. Materials and Methods

### 2.1. Chemicals and Standards

N-hexane, ethyl acetate, acetonitrile, 2-propanol, and methanol were acquired from Sigma-Aldrich (Milan, Italy), toluene was purchased from Romil ltd (Cambridge, UK), anhydrous absolute ethanol and acetic acid were purchased from Carlo Erba Reagents (Milan, Italy). Ultra-pure water was produced by a Milli-Q system (Millipore Corporation, Billerica, MA, USA).

The acronym/name and manufacturing company of compounds sought in this study, including the internal standards, are provided in Table S1 of the Supplementary Material. Stock standard solutions were prepared in methanol at a concentration of 100 µg/mL and stored at −20 °C; working solutions were weekly prepared after a proper dilution.

### 2.2. Materials

Florisil, MgO_3_Si 60–100 mesh was purchased from Sigma-Aldrich S.r.l. (Milano, Italy).

Phenex-RC (Regenerated Cellulose) filters, 15 mm, with a pore size of 0.20 μm were acquired from Phenomenex (Torrance, CA, USA). PVDF (polyvinyldenfluoride) filters, 4 mm (0.22 μm pore size), were acquired from Merck Millipore (Billerica, MA, USA). Filters with a diameter of 25 mm, porosity 0.20 μm in RC (Regenerated Cellulose), were acquired from CPS (Milan, Italy). A 1 mL glass syringe was acquired by Poulten & Graf (Wertheim, Germany).

The GC column was a capillary DB17 (50% fenil-metilpolisilossano (30 m × 0.25 mm), film 0.25 μm, J&W Scientific (Folsom, CA, USA).

The HPLC column was a C_18_ Waters Xbridge BEH (Ethylene Bridged Hybrid) (2.5 μm × 2.1 mm × 100 mm) (Milford, MA, USA).

### 2.3. Samples

**Figure 1:**
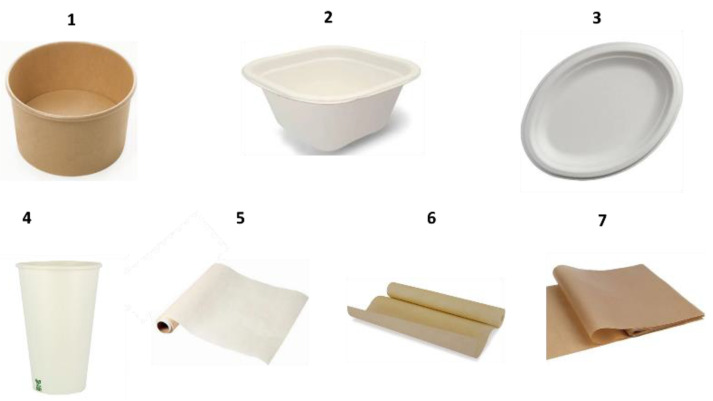
BBFCMs investigated in this work.

In this study we analyzed disposable food contact materials made of sugarcane bagasse or cellulose pulp derived from wood. Items showing the label “*natural, compostable, or biodegradable*” were purchased at supermarkets, fast-food and retail shops. The seven packaging samples analyzed were a food box, a microwave tray, a plate, a cup, and three different types of baking paper, as shown in Figure 1, and these are listed and coded in Table 1.

Although these BBFMCs are composed of natural matter, they still undergo different steps of production, which are not always clearly known. For example, a bleaching process has been declared only for Sample 5, and Sample 7 has been labeled as siliconized; however, any technical specification is missing for the other samples.

### 2.4. Instruments

Reference materials, samples and standards were weighted on an analytical balance (mod. Sartorius BP-211-D). Evaporators (Dionex SE500s, Miulab NDK200-2N) and rotary evaporator (BÜCHI, Villebon sur Yvette, France) were used to dry the handled samples.

PFAS analyses were performed in MRM mode on an ESI-Q-TRAP (SCIEX QTRAP 6500, AppliedBiosystem SCIEX, Darmstadt Germany) coupled to high-performance liquid chromatography (HPLC) (Shimadzu Nexera X2, Reinach, Switzerland) to reach the best selectivity and sensitivity. The data were processed using Analyst software version 1.7.2. The separation was performed in reverse-phase. The mobile phase (flow rate = 0.2 mL min^−1^) used was ammonium acetate 15 mM (MP A) and MeOH (MP B) in a ratio of 45:55, running in gradient mode (see Supplementary Table S2). The analysis time was 20 min, at 35 °C, and the injection volume was 5 µL.

OPE analyses were carried out on both a GC-EI-MS (Agilent. 7890Bs and 5977B, Agilent Technologies Palo Alto, CA, USA) for the most apolar compounds and a HPLC, coupled to an ESI Q-q-Q (Agilent 1290 + Agilent G6460 Agilent technologies Palo Alto, CA, USA), for most polar compounds. Chromatographic and mass spectrometric conditions are reported elsewhere [14,18].

### 2.5. Calibration Curves, Linearity, Limit of Detection and Limit of Quantification

Due to the absence of matrix effect, calibration curves were built in solvent using five calibration levels ranging between 2 and 30 ng/mL for PFASs and between 5 and 100 ng/mL for OPEs. LOD is the lowest concentration that produces an analyte signal in the chromatogram equal to at least three times the background noise, while the LOQ describes the lowest concentration that produces an analyte signal in the chromatogram equal to at least ten times the background noise. To obtain these two parameters, a multistandard solution containing all the reference analytes was used. The measurement was based on the progressive dilution method, evaluating the signals of the analyte in the chromatogram. The intra-day repeatability was calculated on multiple measurements performed on the same standard or sample on the same day. 

This value was expressed as relative standard deviation percentage (RSD%). For inter-day repeatability, three triplicate migration tests on the same sample were carried out in three different days. A value of RSD < 25% was considered acceptable. Inter and intra-day repeatability was calculated for all three OM tests used in this work.

### 2.6. Extraction Protocol for the Total Amount of Contaminants

To optimize the contaminant extraction protocol, tests at high temperature and pressure by Accelerated Solvent Extraction (ASE-200 Dionex, Sunnyvale, CA, USA) [18] and ultrasonic extraction (US Elma Transsonic T 460/H Elma Schmidbauer GmbH Gottlieb-Daimler-Straße 17224 Singen, Germany) were carried out on BBFCMs and compared. 

OPE extraction showed comparable results for both US and ASE, although US was shown to be the most effective method to extract PFASs and was chosen as the best technique to extract organic species from packaging. 

To determine the appropriate size of samples to be used for the extraction of the investigated contaminants, trials were carried out using pieces of 10 × 10 cm^2^ and 2.5 × 2.5 cm^2^. Sample pieces were then put into contact with an equal amount of extracting solvent (20 mL). It was observed that the extraction of contaminants was maximized for a smaller sample size (2.5 × 2.5 cm^2^).

**Figure 2:**
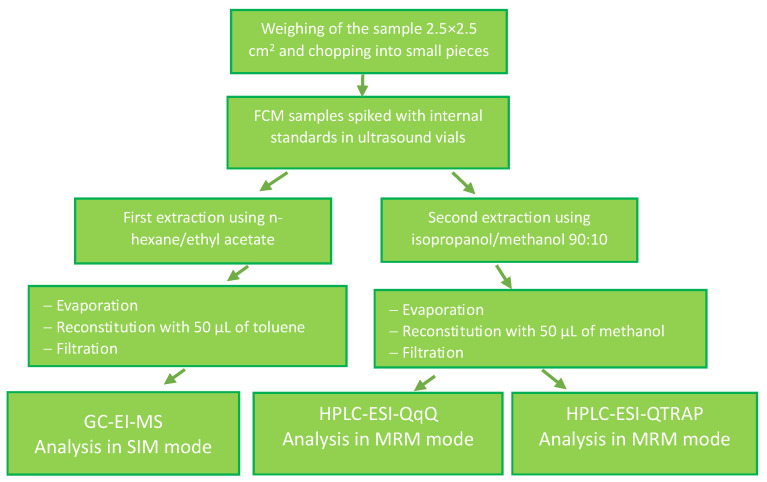
Scheme of the sample extraction to study the total contamination of BBFCMs.

Summing up, the analytical procedure was divided into 3 main steps accounting for sample preparation, US extraction, and solvent evaporation, as shown in Figure 2. The choice of solvents and other details of this procedure are explained elsewhere [14].

### 2.7. Migration Tests

As mentioned, the migration procedure follows the total migration tests approved by European legislation [8]. The two chosen simulants were 95% ethanol *v*/*v* (D2) and 3% *v*/*v* acetic acid (B), simulating fatty foods and acidic aqueous-based foods, respectively.

The standardized testing conditions OM2, OM3, and OM4 shown in Table 2 were applied to the seven BBFCMs by using 2.5 × 2.5 cm^2^ of sample (previously weighed) in contact with 20 mL of the simulant in a test vial. 

## 3. Results

### 3.1. Steps of the Work

For evaluating the total content of the compounds listed in Table S1 and to optimize the analytical procedure for studying the migration of the analytes from the investigated matrices to the food simulants (simulant D2 = 95% *v*/*v* ethanol; simulant B = 3% *v*/*v* acetic acid), the work was carried out in the following phases:

- Optimization of the detection and quantification method for PFASs by HPLC-ESI-Qtrap;
- Construction of calibration curves and determination of the main method validation parameters for PFASs (LOD and LOQ, linearity and inter- and intra-day repeatability);
- Evaluation of the total content of contaminants and migration of OPEs and PFASs in seven samples of biodegradable, compostable or recyclable BBFCMs.

#### 3.1.1. Mass Spectrometric Parameters for PFASs

First, we optimized the mass spectrometric parameters on an ESI-Q-trap instrument by the infusion of individual compounds at a flow rate of 5 μL/min and a concentration of 500 ng/mL, performing MS and MS-MS spectra. The most significant transitions were chosen for subsequent HPLC-MS/MS analysis in MRM mode. The gradient elution used is reported in Tables S2 and S3 showing precursor ions, product ions, declustering potential (DP), collision energy (CE), retention times (tR) and five acquisition windows. The qualifier ions are reported in brackets. 

**Figure 3:**
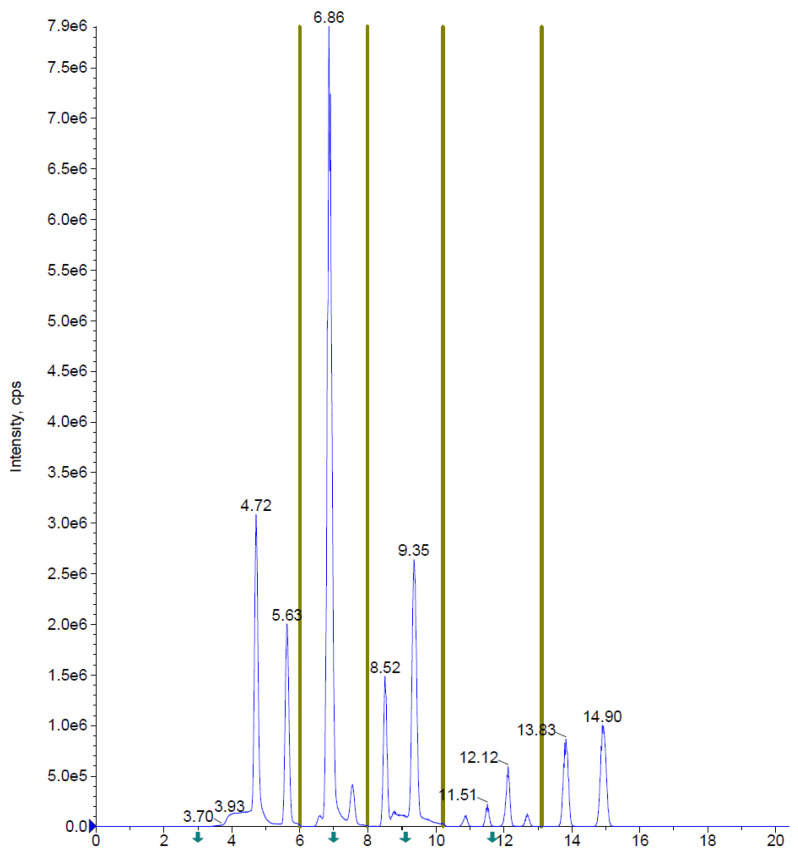
HPLC-ESI-Qtrap analysis in MRM mode of PFASs multistandard mixture (50 ng/mL), HPLC and mass spectrometry conditions as in Section 2.4 and in Tables S2 and S3.

Some of the molecules showed very close retention times, but the coupling to mass spectrometry allowed us to separate, thanks to the different transitions, the signals of the co-eluting analytes. Figure 3 shows a separation carried out by HPLC-ESI-Qtrap acquisition in MRM mode of a standard mixture of PFASs (50 ng/mL). The identity of the peaks is reported in Table S3 corresponding to the retention times.

#### 3.1.2. Data Quality for PFASs

The coefficients of determination R^2^ of the calibration curves, ranging between 0.997 and 0.999, were used to assess the linearity. The intra-day repeatability ranged between 1 and 25%. LOD and LOQ were evaluated using triplicate analysis of a procedural blank. LODs and LOQs were in the ranges 0.005–1 ng/mL and 0.02–3 ng/mL, respectively (shown individually in Table S4). By considering the final volume of 50 µL of the solution after the evaporation, and the initial weight of each processed sample, the LOD and LOQ ranges are 0.001–1.931 µg kg^−1^ and 0.004–5.792 µg kg^−1^, respectively. 

#### 3.1.3. Total Content of Contaminants and Their Migration from Investigated BBFCMs

##### Total Content of Contaminants 

The total amount of the targeted contaminants present in the seven investigated samples was measured by using the procedure shown in Figure 2. The results of the measurements, made in triplicate to verify reproducibility of the extraction method, are shown in Table 3.

The data show that the most contaminated samples, by both OPEs and PFASs, are the baking papers (samples 5–7). The level of PFASs in all the BBFCMs is comparable to the OPEs concentration except for samples 1 and 7, which show an amount of PFASs that is about three times higher than the OPEs content. 

Figure S1 shows the distribution of individual OPEs within the various BBFCMs. Four of them stand out. Tributoxyethyl phosphate (TBEP) is toxic, as it is known to cause an inhibition of cell profiling. Tributyl phosphate (TBP) is classified as carcinogen for rats via the oral route, while studies are being carried out for triisobutyl isomer (TIBP), which based on its isomeric structure could give rise to similar outcomes [19,20,21]. Lastly, trichlorophenyl phosphate (TCPP), which is also potentially carcinogenic, is present at a relevant percentage.

In regard to PFASs, only 6 of the 23 compounds analyzed were always present in all samples. In addition to PFOA found at concentrations ranging between 0.004 and 0.099 mg kg^−1^, PFBA, PFPeA, PFHxA, PFHpA, and PFDoS were found at concentrations of the same order of magnitude as PFOA.

##### Migration of Contaminants

For each class of contaminant, the migration of all analytes (sum of all migrated compounds by class) was compared with the corresponding amount of the extracted analytes. The percentage of migration was evaluated according to Formula (1)

(1) $$Migration\;\%=\frac{{\frac{mg}{kg}}_{migrated}}{{\frac{mg}{kg}}_{extracted}}\times 100$$

Within the same class of compounds, some were more likely to migrate. Therefore, for each group, the most representative compounds were evaluated sample by sample in order to estimate the greatest contribution to migration.

### 3.2. OPE Migration

**Figure 4:**
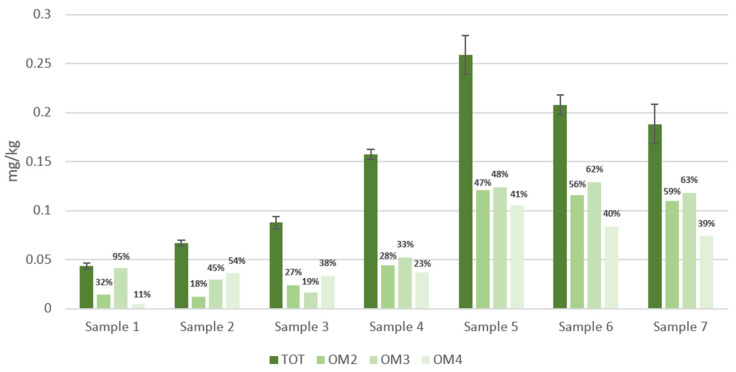
OPE concentrations extracted from seven BBFCMs (TOT) and concentrations of migrated OPEs into 95% ethanol *v*/*v* (simulant D2) after treatment under three different conditions (OM2, OM3, OM4) (data labels display the migration percentage).

Figure 4 shows the total concentration (mg/kg) of OPEs that migrated into ethanol from samples 1 to 7 after the three tests (OM 2, 3, 4) compared to the total extract. The repeatability of the tests was good, since the standard deviation RSD of the triplicate trials was <12%. Percentages of migration, calculated according to Formula (1) and listed in the figure labels, show that the baking papers are the most contaminated samples; in particular, the amount of migrated OPEs exceeds by an order of magnitude the precautionary limit of 0.01 mg kg^−1^.

Furthermore, the graph shows that test OM4 (1 h at 100 °C), in most of the cases, is the least effective in terms of migration compared to tests with a longer contact time.

On average, each sample shows that OPE migration does not undergo significant differences when the applied test varies. We can observe that the percentage of migration in the OM test 3 is drastically higher (95%) than the ones in OM tests 2 (32%) and 4 (11%) only for Sample 1. 

Therefore, the temperature increasing up to 70 °C and the duration of the test for this packaging material has an important effect.

**Figure 5:**
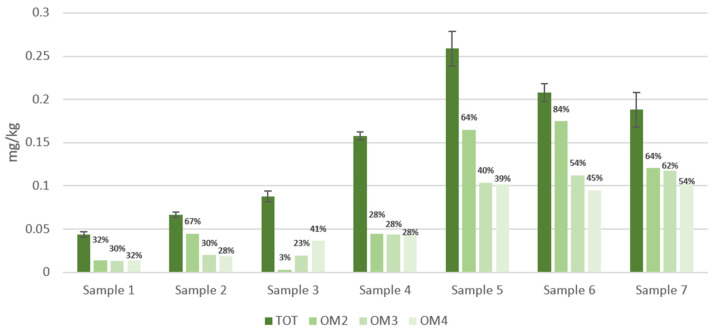
OPE concentrations extracted from seven BBFCMs (TOT) and concentrations of migrated OPEs into 3% *v*/*v* acetic acid (simulant B) after treatment under three different conditions (OM2, OM3, OM4) (data labels display the migration percentage).

Figure 5 shows the total concentration (mg/kg) of OPEs that migrated into 3% *v*/*v* acetic acid from samples 1 to 7, after the three tests (OM 2, 3, 4) compared to the total extract. Regarding the second simulant, the data show that the first test, 10 days at 40 °C, is generally the most effective in terms of migration, whereas the other two tests show similar results. Probably, in relation to this simulant, the contact time is a more important variable than the temperature. In this case, again, the sum of the migrated OPEs in each test is above the precautionary limit set at 0.01 mg/kg.

About the distribution of the analytes in both simulants, tributoxyethyl phosphate (TBEP), triisobutyl phosphate (TIBP), tris (1-chloro-2-propyl) phosphate (TCPP), and, to a lesser extent, tributyl phosphate (TBP) are the ones that mainly contribute to the total OPEs content. TIBP is an isomer of TBP whose mutagenic activity has been ascertained.

The carcinogenic TCPP has been included in the fourth list of priorities of the European Commission. These compounds, dangerous to human health, exceed the limit of 0.01 mg/kg. It is interesting to note how TBEP, present at non-negligible concentrations in the packaging materials analyzed, shows a prevalent migration toward simulant B. TIBP and TCPP rather migrate toward simulant D1, also causing a concentration above 0.01 mg/kg.

### 3.3. PFAS Migration

**Figure 6:**
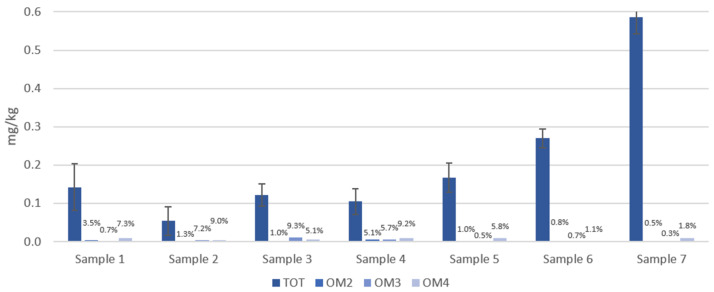
PFAS concentrations extracted from seven BBFCMs (TOT) and concentrations of migrated PFASs into 95% ethanol *v*/*v* (simulant D2) after treatment under three different conditions (OM2, OM3, OM4) (data labels display the percentage of migration).

**Figure 7:**
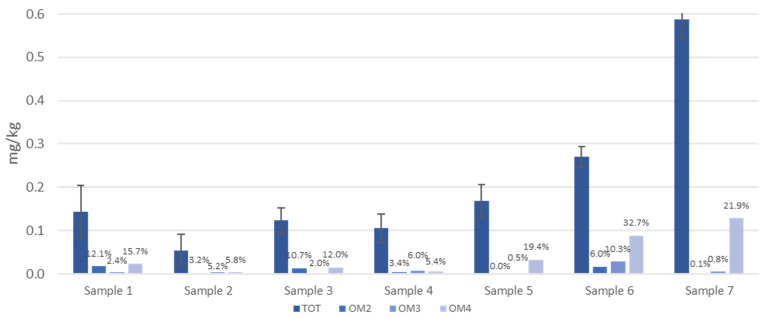
PFAS concentrations extracted from seven BBFCMs (TOT) and concentrations of migrated PFASs into 3% *v*/*v* acetic acid (simulant B) after treatment under three different conditions (OM2, OM3, OM4) (data labels display the migration percentage).

Figure 6 and Figure 7 show the total concentration (mg/kg) of PFASs that migrated into ethanol (Figure 6) and acetic acid (Figure 7) from samples 1 to 7 after the three tests (OM 2, 3, 4) compared to the total extract. 

It is readily noticeable that the migration of PFASs is much lower than that of OPEs (below 10% in ethanol and never higher than 33% in acetic acid). The acetic acid simulant seems to be more effective, especially in the OM4 test, which is a sign that high temperatures positively influence PFAS migration, especially from the three baking papers (samples 5, 6 and 7) and the food box (sample 1) toward aqueous, acidic food.

Since the migration of PFAS is lower than that of the OPEs, the concentration of migrated OPEs in both simulants is at least one order of magnitude greater than that of PFAS, except for test OM4 simulant B, where the PFAS and OPE concentrations are comparable and higher than 0.01 mg/kg. The PFAS responsible for elevated concentrations is essentially PFBA (see Supplementary Material Figure S3), never exceeding a concentration of 0.12 mg/kg. Nevertheless, this result is not cause of concern since experimental evidence has indeed demonstrated a possible PFBA toxicity for humans at exposure levels ≥ 30 mg/kg body weight per day [22]. The second most abundant PFAS is PFHxA (see Supplementary Material Figure S3). The detected PFASs are both short-chain compounds (4–6 carbon atoms, respectively) and able to easily migrate in a simulant such as acetic acid 3%. PFHxA is a substance of Very High Concern for being persistent, bioaccumulative and toxic (PBT). 

## 4. Conclusions

Bio-based food packaging materials labeled as natural, compostable or biodegradable can anyway imply the use of additives necessary to increase rigidity, flexibility, water and grease proofing.

This explorative study, on the wake of a previous one, has shown that two classes of contaminants, OPEs and PFASs (the latter known as Forever Chemicals), are present in material of natural origin and can migrate from bio-based food contact materials into simulants and hence to food. The presence of the so-called Forever Chemicals in the investigated materials should be a matter of concern due to potential environmental implications. The migration by contact of all substances known as toxic has human health implications.

The two classes of contaminants were first investigated in the biodegradable and compostable packaging matrices. Afterwards, since the extent of migration from FCM to food depends on several factors, including the type of compound, the temperature, and the contact time, the percentages of migration from these matrices toward two food simulants, D2 (95% ethanol which mimics the behavior of fatty foods) and B (3% acetic acid which mimics the behavior of acid-aqueous foods) were evaluated according to the European directives.

In particular, the present work has proposed a quantification method for PFASs by the use of the highly sensitive HPLC-QTRAP-MS/MS technique. The optimization of an analytical method with high instrumental sensitivity is necessary to analyze PFASs, since a significant and expansive restriction proposal in the EU has begun its process toward the promulgation of the law and fluorocompounds will soon be totally banned from food contact materials. 

Furthermore, the methods already in use in the laboratory, for the analysis of polar OPEs by HPLC-QqQ, and apolar OPEs by GC-EI/MS, were confirmed.

The results showed that the different packaging matrices are contaminated by a comparable content of OPEs and PFASs that only exceed OPEs in samples 1 and 7. The most contaminated matrices were baking papers. TIBP and TCPP, among the best known OPEs for their toxicity, exceed the limit of 0.01 mg/kg. The results obtained with the use of food simulants demonstrate that OPEs migrate in a comparable manner toward both food with hydrophilic and lipophilic properties, while PFASs mainly migrate toward aqueous acid food due to their more polar nature. 

This exploratory work demonstrates that the monitoring of pollutants in food packaging should also be carried out on bio-based packaging from biodegradable sources, which may cause concern for human health, despite being *greener* alternatives. Future developments of the work may include the application of OM TEST present in the European legislation to a wider range of contaminants in order to have a more realistic general overview. Furthermore, the range of packaging investigated will need to be expanded and the migration of contaminants into real foods will need to be assessed and quantified.

## Figures and Tables

**Table 1:** Code and kind of the investigated sample.

Code	Food Contact Material
Sample 1	Food box
Sample 2	Microwave tray
Sample 3	Plate
Sample 4	Cup
Sample 5	Baking paper A
Sample 6	Baking paper B
Sample 7	Baking paper C

**Table 2:** Migration test (OM) reported in (EU) No 10/2011 [8] applied to the samples.

Standardized Test Conditions		
Column 1	Column 2	Column 3
**OM2**	10 d at 40 °C	Any long-term storage at room temperature or below, including heating up to 70 °C for up to 2 h or heating up to 100 °C for up to 15 min.
**OM3**	2 h at 70 °C	Any contact conditions that include heating up to 70 °C for up to 2 h or up to 100 °C for up to 15 min, which are not followed by long-term storage at room or refrigerated temperature.
**OM4**	1 h at 100 °C	High-temperature applications for all types of food at temperature up to 100 °C.

**Table 3:** Concentrations (mg/kg) and relative standard deviation of PFASs and OPEs in different BBFCMs.

	Sample 1	Sample 2	Sample 3	Sample 4	Sample 5	Sample 6	Sample 7
OPEs (mg/kg)	0.04 ± 0.01	0.07 ± 0.01	0.09 ± 0.01	0.16 ± 0.01	0.26 ± 0.06	0.21 ± 0.01	0.19 ± 0.02
PFASs (mg/kg)	0.14 ± 0.06	0.05 ± 0.04	0.10 ± 0.03	0.11 ± 0.05	0.17 ± 0.04	0.27 ± 0.05	0.59 ± 0.04

## Data Availability

The original contributions presented in the study are included in the article/supplementary material, further inquiries can be directed to the corresponding author

## Supplementary Materials

The following supporting information can be downloaded at https://www.mdpi.com/article/10.3390/foods13050645/s1, Table S1. 22 PFASs and 12 OPEs compounds, 9 Internal Standards, their acronymous, Molecular formula, Molecular Weight and manufacturing company. Table S2. HPLC gradient elution of PFASs. Table S3. Precursor and product ions, electrical parameters (CE, DP, EP, CPX), Tr, time acquisition windows of PFAS analytes in HPLC-ESI-QTRAP. Qualifiers ions are in brackets. Table S4: Validation parameters. Figure S1. Main OPEs extracted from the different BBFCMs. Figure S2. Main PFASs extracted from the different BBFCMs. Figure S3. Main PFASs migrated in simulant B from 1,5,6 and 7 BBFCMs.
