# Supplementary material for: Studies of Potential Migration of Hazardous Chemicals from Sustainable Food Contact Materials
Source: Foods. 2024 Feb 21;13(5):645. doi: 10.3390/foods13050645 (PMC10930497; doi:10.3390/foods13050645)
Supplement: Supplementary file 1 [file foods-13-00645-s001.zip › foods-2866501-Supplementary material.pdf]

Table S1. 22 PFASs and 12 OPEs compounds, 9 Internal Standards, their acronymous, Molecular formula, Molecular Weight and manufacturing company.

Name	Acronymous	Molecular Formula	Molecular Weight (g/mol)	Manufacturing Company
Perfluorobutanoic acid	PFBA	C_3F_7COOH	214. 0	Wellington Laboratories (Ontario, Canada)
Perfluoro-n-$[^{13}C_4]$ butanoic acid	m-PFBA	$[^{13}C]_4F_7OOH$	218. 0	
Perfluoro-n-pentanoic acid	PFPeA	C_4F_9COOH	264.1	
Perfluorobutanesulfonic acid	PFBS	$C_4F_9SO_3H$	300.1	
Perfluorohexanoic acid	PFHxA	$C_5F_{11}COOH$	314.1	
Perfluoro-n-$[^{13}C_2]$hexanoic acid	m-PFHxA	$[^{13}C]_2C_3 F_{11}COOH$	316.1	
Hexafluoropropylene oxide-dimer acid	GenX	$C_6H_4F_{11}NO_3$	347.1	Apollo Scientific (Regno Unito)
Perfluoropentanesulfonic acid	PFPeS	$C_5F_{11}SO_3H$	350.1	Wellington Laboratories (Ontario, Canada)<br>Wellington Laboratories (Ontario, Canada)
Perfluoro-n-heptanoic acid	PFHpA	$C_6F_{13}COOH$	364.1	
Perfluorohexanesulfonic acid	PFHxS	$C_6F_{13}SO_3H$	400.1	
Sodium perfluoro-1-$[^{13}C_3]$hexanesulfonate	m-PFHxS	$C_3[^{13}C]_3F_{13}SO_3H$	403.1	
Perfluorooctanoic acid	PFOA	$C_7F_{15}COOH$	414.1	
Perfluorooctanoic acid -$[^{13}C_4]$	m-PFOA	$C_4[^{13}C]_4HF_{15}O_2$	418.1	
Perfluoroheptanesulfonic acid	PFHpS	$C_7F_{15}SO_3H$	450.1	
Perfluorononanoic acid	PFNA	$C_8F_{17}COOH$	464.1	
Perfluoro-n-$[^{13}C_5]$nonanoic acid	m-PFNA	$[^{13}C]_5\ C_3F_{17}COOH$	469.1	
Perfluorooctanesulfonic acid	PFOS	$C_8F_{17}SO_3H$	500.1	
Sodium perfluoro-1-$[^{13}C_4]$octanesulfonate	m-PFOS	$[^{13}C]_4\ C_4F_{17}SO_3H$	504.1	
Perfluorodecanoic acid	PFDA	$C_9F_{19}COOH$	514.1	
Perfluorononanesulfonic acid	PFNS	$C_9F_{19}SO_3H$	550. 1	

Perfluoroundecanoic acid	PFUnDA	$C_{10}F_{21}COOH$	564.1	
Perfluoro-n-[$^{13}C_7$]undecanoic acid	m-PFUnDA	$C_3[^{13}C]_7F_{21}COOH$	571.1	
Perfluorodecanesulfonic acid	PFDS	$C_{10}F_{21}SO_3H$	600.2	
Perfluoro-n-dodecanoic acid	PFDoA	$C_{11}F_{23}COOH$	614.1	
Perfluorotridecanoic acid	PFTrDA	$C_{12}F_{25}COOH$	664.1	
Perfluorodecane sulfonic acid	PFDoS	$C_{12}F_{25}SO_3H$	700.2	
Perfluorotetradecanoic acid	PFTeDA	$C_{13}F_{27}COOH$	714.1	
Perfluorohexadecanoic acid	PFHxDA	$C_{15}F_{31}COOH$	814.1	
Perfluoro-n-octadecanoic acid	PFODA	$C_{17}F_{35}COOH$	914.1	
Triethyl phosphate	TEP	$C_6H_{15}PO_4$	182,2	
Tripropyl phosphate	TPrP	$C_9H_{21}PO_4$	224,2	Sigma-Aldrich S. r. l. (Milano, Italia)
Tributyl phosphate	TBP	$C_{12}H_{27}PO_4$	266,3	
Tri-*iso*butyl phosphate	TIBP	$C_{12}H_{27}O_4P$	266,3	
Tris(2- chloroethyl) phosphate	TCEP	$C_6H_{12}PO_4Cl_2$	285,5	
Tris(2- chloroethyl) phosphate -d_{12}	TCEP-d_{12}	$(ClCD_2CD_2O)_3PO$	297,5	
Triphenyl phosphate	TPhP	$C_{18}H_{15}PO_4$	326,3	
Triphenyl phosphate-d_{15}	TPhP-d_{15}	$(C_6D_5O)_3PO$	341,4	
2-ethylhexyl diphenyl phosphate	EHDPhP	$C_{20}H_{27}PO_4$	362,4	Wellington Laboratories (Ontario, Canada)
tri-m-cresyl phosphate	TMTP	$C_{21}H_{21}PO_4$	368,4	
Tris(2-chloroisopropyl) phosphate	TCPP	$C_9H_{18}C_{l3}O_4P$	327,57	
Tris(2-butoxyethyl) phosphate	TBEP	$C_{18}H_{39}PO_7$	398,5	

Tris(1,3-dichloro-2-propyl) phosphate	TDCPP	$C_9H_{15}PO_4Cl_6$	431,3	
Tris(2-ethylhexyl) phosphate	TEHP	$C_{24}H_{51}PO_4$	435,0	

Table S2. HPLC gradient elution of PFASs

Time (min)	MP A 15mM CH_3COONH_4	MP B MeOH
0.10	45	55
10	1	99
20	1	99
20.01	90	10

Table S3. Precursor and product ions, electrical parameters (CE, DP, EP, CPX), Tr, time acquisition windows of PFAS analytes in HPLC-ESI-QTRAP. Qualifiers ions are in brackets.

Analyte	Precursor ion (m/z)	Product ion (m=z)	CE (eV)	DP (V)	EP (V)	CPX (V)	Tr (min)	Time acquisition window (min)
PFBA	213	169	-14	-10	-10	-11	4.21	0-5.5.5
m-PFBA	217	172	-14	-10	-10	-11	4.21	
PFPeA	263	219	-14	-10	-10	-11	5.07	
PFBS	299	98.6	-38	-50	-10	-11	5.11	
PFHxA	313	119	-38	-10	-10	-11	6.06	5.5/7.4
m-PFHxA	315	119	-38	-10	-10	-11	6.05	
PFPeS	349	99	-25	-60	-10	-11	6.07	
genX	285	187	-25	-60	-10	-11	6.36	
genX(q)	285	169.2	-25	-60	-10	-11	6.36	
PFHpA	363	169.0	-25	-10	-10	-11	7.07	
PFHpA(q)	363	319	-25	-10	-10	-11	7.07	
PFHxS	399	99	-20	-53	-10	-11	7.04	
PFHxS(q)	399	80	-20	-53	-10	-11	7.04	
m-PFHxS	403	103	-20	-53	-10	-11	7.04	
m-PFHxS(q)	403	84	-20	-53	-10	-11	7.04	
PFOA	413	369	-20	-10	-10	-11	8.05	7.4/8.37
PFOA(q)	413	169	-20	-10	-10	-11	8.05	
m-PFOA	417	171.9	-25	-10	-10	-11	8.04	
m-PFOA(q)	417	371.9	-25	-10	-10	-11	8.04	
PFHpS	449	80	-20	-53	-10	-11	7.98	

PFHpS(q)	449	99	-20	-53	-10	-11	7.98	
PFNA	463	419	-20	-10	-10	-11	8.94	8.37/12.31
PFNA(q)	463	169	-20	-10	-10	-11	8.94	
m-PFNA	468	423	-20	-10	-10	-11	8.94	
m-PFNA(q)	468	169	-20	-10	-10	-11	8.94	
PFOS	499	79.8	-120	-117	-12	-11	8.86	
PFOS(q)	499	99	-120	-117	-12	-11	8.86	
m-PFOS	503	79.8	-120	-117	-12	-11	8.87	
PFNS	549	99	-43	-57	-10	-11	9.63	
PFDA	513	169	-43	-10	-10	-11	9.70	
PFDA(q)	513	469	-43	-10	-10	-11	9.70	
PFTeDA	712	669	-35	-10	-10	-15	12.11	
PFDS	599	89	-43	-57	-10	-11	10.31	
PFDS (q)	599	99	-43	-57	-10	-11	10.31	
PFUnDA	563	269	-18	-10	-10	-11	10.41	
m-PFUnDA	570	276	-18	-10	-10	-11	10.41	
PFDoA	613	269	-28	-10	-10	-11	11.04	
PFDoS	699	80	-50	-57	-10	-11	11.42	
PFDoS (q)	699	99	-50	-57	-10	-11	11.42	
PFTrDA	663	619	-30	-30	-10	-11	11.50	
PFTrDA(q)	663	169	-30	-30	-10	-11	11.50	
PFTeDA	712	669	-35	-10	-10	-16	12.11	
PFHxDA	813	769	-38	-200	-10	-11	13.34	12.66/30
PFHxDA(q)	813	269	-38	-200	-10	-11	13.34	
PFODA	913	869	-38	-200	-10	-11	14.63	

Table S4: Validation parameters

Compound	R^2	Intra-day Repeatability (RSD%)	Inter-day Repeatability (RSD%)	LOD/LOQ (ng/mL)
PFBA	0.9915	12	15	0.005-0.02
PFPeA	0.9941	4	6	0.005-0.02
PFBS	0.9929	13	18	0.005-0.02
PFHxA	0.9867	5	8	0.005-0.02
GenX	0.9875	6	9	0.005-0.02
PFPeS	0.9941	9	12	0.5-2
PFHpA	0.9894	10	15	0.005-0.02
PFHxS	0.9887	5	7	1-3
PFOA	0.9947	4	6	0.005-0.02
PFHpS	0.9814	6	8	1-3
PFNA	0.9942	3	5	0.005-0.02
PFOS	0.9900	4	8	0.005-0.02
PFDA	0.9853	9	13	0.5-2
PFNS	0.9817	7	10	0.5-2

PFUnDA	0.9986	4	8	0.5-2
PFDS	0.9926	8	11	0.5-2
PFDoA	0.9899	4	7	0.5-2
PFTrDA	0.9901	18	24	0.1-0.3
PFDoS	0.9898	15	20	1-3
PFTeDA	0.9903	14	18	0.1-0.3
PFHxDA	0.9958	12	16	0.05-0.2
PFODA	0.9999	9	12	1-3

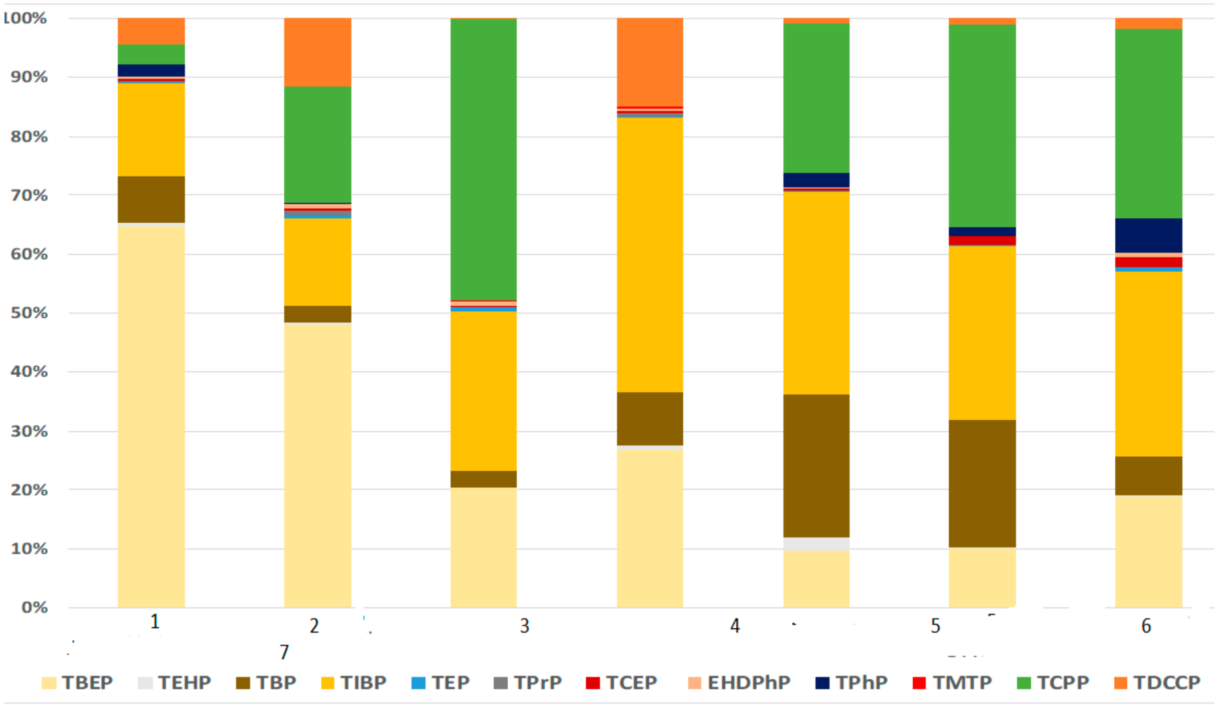

Figure S1. Main OPEs extracted from the different BBFCMs

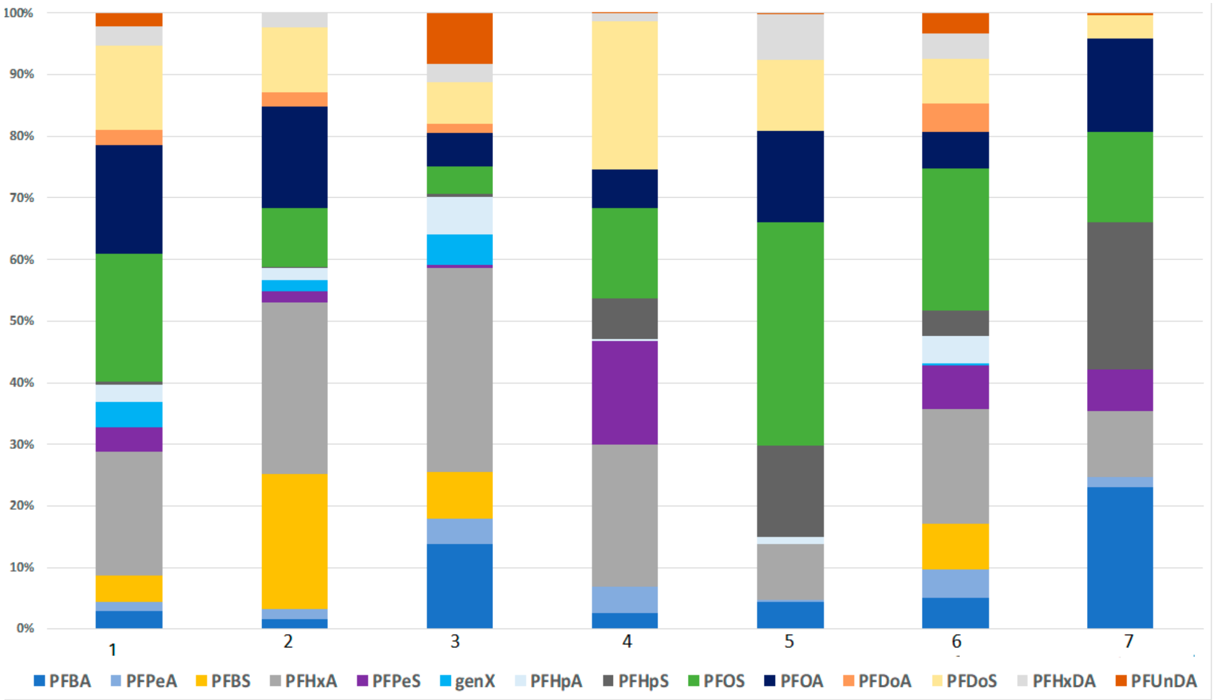

Figure S2. Main PFASs extracted from the different BBFCMs

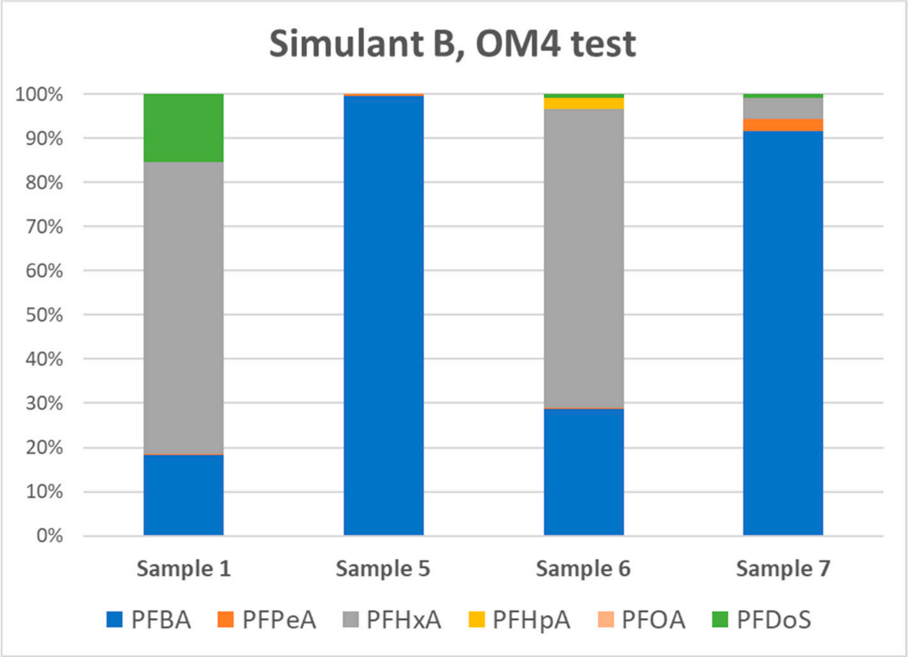

Figure S3. Main PFASs migrated in simulant B from 1,5,6 and 7 BBFCMs
